# A qualitative analysis of the effectiveness of telehealthcare devices (i) are they meeting the needs of end-users?

**DOI:** 10.1186/s12913-017-2408-8

**Published:** 2017-07-04

**Authors:** Natasha C. Campling, David G. Pitts, Paul V. Knight, Richard Aspinall

**Affiliations:** 1 Faculty of Health Sciences Building 67, University of Southampton, Southampton, UK; 2 Cranfield Biotechnology Centre, Cranfield University, Bedford, UK; 3 Health and Wellbeing Academy, Anglia Ruskin University, Bishop Hall Lane, Chelmsford, CM1 1SQ UK

**Keywords:** Telecare, Telehealth, Devices, End-users, Effectiveness, Needs

## Abstract

**Background:**

There are many telehealthcare devices currently available ranging from personal alarms, automated pill dispensers and fall detectors through to monitoring devices for blood sugar, blood pressure and heart rate. Many devices remain unused once acquired or shortly after a period of initial use.

**Methods:**

The study used a qualitative design involving focus groups and interviews. End users’ opinions of telehealthcare devices were examined through focus groups along with the views of market experts and key supply chain players through telephone interviews to ascertain their views on the devices. The data were recorded, transcribed and analysed thematically.

**Results:**

Amongst the wide range of user issues associated with telehealthcare devices two themes merited particular attention: design characteristics and the lack of focus on end-user needs. Our findings suggested that few telehealthcare devices appear to be developed based on the principles of user-centred design. Consequently, many were non-intuitive to use, with the majority of the focus group participants not recognising the purpose of the devices from their appearance alone.

**Conclusions:**

Greater input from real end-users rather than “proxy” users such as carers, professional users or technologists is required when developing telehealthcare devices or systems. Design should be focussed on intuitive use to enable the user to successfully achieve what is required from the devices. This may require the existing supplier—driven market focus to be challenged, but could improve the contribution of technology to improving healthcare.

## Background

In 2012 when the global population reached 7 billion, 562 million of those individuals were aged 65 or over. This constituted approximately 8% of the population [1]. By 2016 this 65 and older population grew to be 9% of the overall population. But in that 4 year period the overall population had risen by over 330 million people, so the 65 and over population now numbers over 635 million [2]. Within this older population the greatest increase in numbers has been seen within the oldest old (85+). These individuals are often considered to be distinct as prolific users of health and social care services. The rates of emergency admission within this group was shown recently to be almost 10 times higher than the section of the population aged between 20 and 49 in England [3]. Comorbidities are common in this oldest old population with suggestions that women in this group show higher disease counts and disability scores than men but most members of this group show long-term conditions needing monitoring and long term care [4]. Such an increase in numbers living with comorbidities places the current health and social care systems under unprecedented demand [5]. This demand is likely to only increase with time causing costs to rise and arguably the quality of care to diminish [6].

The budget for the NHS in England for 2015/16 is £116.4 billion [7] and more than 40% of national health spending is required for people over the age of 65 [5]. Those older individuals in society cost more with estimates from the Nuffield Trust that those over 85 each cost the NHS about £7000 per year [5]. Technology is often viewed as a mechanism to reduce the costs associated with this care and telehealthcare devices are seen as a means to keep individuals independent at home for as long as possible whilst reducing demands on primary and secondary care where appropriate. This use of technology as an enabler to improve healthcare has grown without close restraint leading to some confusion in the terms used to define activities. These terms include: telemedicine and telerehabilitation (conventional medicine delivered at a distance), telehealth (remote collection of patient data), telecare (personal and environmental sensors used to detect risks or events), telecoaching (a preventive approach focused upon behavioural change), MHealth (mobile health), including self-care apps (health, care and wellbeing applications available for use on internet-enabled mobile devices) [6], Ehealth (electronic health) and DHealth (digital health) (terms that cover all aspects of the intersection of health and ICT) [8]. This paper uses the comprehensive term ‘telehealthcare’ to encompass both telehealth and telecare provision.

Whilst many technologies and devices have been designed to enhance the ability of older populations to manage living at home for longer, their design and production has not always included end user involvement. User involvement during the initial phases of any product development is regarded by many as important as it helps capture user needs, which benefits both users and producers and improves usage. Consequently there are numerous barriers to usability and ultimately device adoption. With this in mind we aimed to determine through focus groups with users and potential users, and interviews with experts within the homecare telehealthcare sector, the range of views on existing products from pendant alarms through to blood pressure monitors and tracking devices.

## Methods

The study utilised focus groups with users and telephone interviews with experts with experience of telehealthcare devices and the telehealthcare sector. Both approaches were carried out after ethical approval.

### Focus group participants and design

The inclusion and exclusion criteria for the focus groups were broad to reduce the potential for bias. Participants for focus groups were recruited from different geographical locations (Bedfordshire and Oxfordshire) and sought through local group organisations. Individuals interested in taking part were contacted and provided with more details of the study. If the individual wished to take part consent forms were sent either via post or email. Fifteen participants took part in the Bedfordshire focus group (with no non-attendees). The group was balanced for gender, with eight participants being male and seven female. Their ages ranged between 65 and 84 years of age, four being between 65 and 69, three between 70 and 74, five between 75 and 79 and three from 80 to 84. In terms of health problems, four stated that they had mobility issues (one used a mobility scooter). One participant was using a pendant alarm (supplied via social services and operated via the local council). The majority (with a sole exception) owned a mobile phone and all were using computers/tablets and email. A number of the group also had access to blood pressure monitors at home, which had been privately purchased. The group in Oxfordshire also saw high attendance with 12 of the 13 participants originally recruited attending. Eleven of the group were female and one male. The age range was wider than with the Bedfordshire group, from 60 to over 85 years of age. Two were aged between 60 and 64, two aged 70-74 and a further two were 75-79. The remaining half of the group were aged over 80 years old (four were aged 80-84, and two were aged 85 or above). Perhaps as a result of this older demographic the health needs of this group were greater. One lived in sheltered housing, three stated that they had joint pain or mobility issues, and three others stated more complex health needs related to Parkinson’s, cancer and an autoimmune condition. The majority owned mobile phones, two of which had purchased big button mobiles with an emergency (SOS) button on the back. Three were using pendant alarms, one couple owned a blood pressure monitor and all were using computers/tablets and email.

### The devices

A number of available devices were introduced sequentially in six groups (Table 1) and displayed in order to stimulate views and thoughts from the participants. Devices were chosen because they identified or aided common issues amongst older individuals, such as; fall detection often linked to location sensing, medication (pill) dispensing and vital sign monitoring. Prior to outlining each group of devices, the participants were asked what they thought was the function of the device.

**Table 1:** Groups of devices used in the study

Group 1: Telecare base units and associated sensors	
Tynetec Reach	Home base unit and sensor hub
Tunstall Lifeline Vi	Home base unit and sensor hub
Bogus caller/intruder alarm	Door entry alarm
Passive infra-red sensor	Room occupancy/motion sensor
Group 2: Pill dispensers	
Pivotell – Altec 169 MHz	Automated dosette box for dispensing medication. Can relay data to home base unit
Group 3: Personal alarm types	
Alarm (basic) e.g. Tunstall MyAmie, Amie+	Wrist worn, pendant or brooch
Group 4: Falls monitor	
Tunstall	Atmospheric pressure + accelerometer, includes manual alarm
Group 5: Mobile alarms/GPS tracking devices	
GeoCare	Tracking device, alarms sent to designated personal number(s), no falls detection
MobileHelp	Mobile personal alarm, no falls detection
buddi	Personal alarm, falls detection, GPS tracking, geofencing
Numera Libris	Speakerphone to response centre, falls detection, tracking
Group 6 Telehealth/ measurement of physical functioning	
BP monitors	Electronic blood pressure monitors with digital displays
Pulse oximeter	Finger-based probe connected to hand-held monitor providing digital display of heart rate and blood oxygen levels
Ear thermometer	Digital tympanic membrane thermometer

### Focus group data analysis

The focus group discussions were audio-recorded via a digital recording device (Philips DVT7000) with an omni-directional table-top microphone. Recording allowed for the discussion to be transcribed verbatim. The data obtained from the group transcripts were handled within NVivo 10 (a qualitative data analysis software package) and analysed using a thematic approach [9].

### The interviews

Preliminary findings from the user focus groups regarding their views on telehealth care devices indicated the need to map the supply chain of these devices. A comprehensive understanding of other stakeholder views was sought in order to help explain why there are disconnections in terms of what users want. As a result, individuals who represented supply chain groups in this area were targeted. The relevant sample groups were: professional bodies, user groups, regulators, providers, county councils, charitable bodies, manufacturers and distributors, research funders and organisations, and trade associations. Those in senior management roles, such as Chief Executives or equivalent were contacted wherever possible, as experts in their fields, who would be willing to speak on behalf of their respective organisations. Suitable candidates were identified via extensive Internet searching and snowball sampling. Potential participants were invited to take part via email. To maintain anonymity those who participated have been placed into the sample groups outlined in Table 2.

**Table 2:** The interview sample groups

Sample group	Interviewees
User groups	2
County Council related	4
Research related organisations	7
Private company specialists	4
Manufacturers and/or distributors, including: Telecommunications industry Trade associations	4
Health related, including professional bodies	6
Total:	27

### Interview guide

Interviews were conducted using a semi-structured guide covering the following areas:

- What are your views regarding the current uptake of telecare/telehealth devices?
- What are the barriers to device uptake?
- What are your views regarding user involvement in device development?
- What are your views on access and supply of devices?
- How can device uptake be promoted in the future?

The interviews ranged in length from between 30 min to an hour and a quarter. In total 27 interviews were conducted with experts; individuals were no longer approached once theoretical saturation had been achieved in the respective sample groups. Data collection and analysis occurred simultaneously so as to test recurrent themes arising from the analysis (constant comparative analysis [10]).

### Interview data analysis

All the interviews were audio-recorded and transcribed verbatim. As with the data from the focus groups, the interview data were handled within NVivo 10 and analysed using a thematic approach [9]. Thematic analysis was selected as the method of analysis for the interview and focus group data as it enabled patterns (themes and resulting categories) across the two data sets to be constantly compared and drawn together to describe the experience of end-users in relation to the usability of telehealthcare devices. The analysis was performed through coding in phases to view meaningful patterns across the data. The phases of analysis were: familiarisation with the data, generation of initial codes, searching for themes amongst codes, reviewing of themes, appropriately defining and naming themes, and writing up findings.

## Results

The findings from both the interview and focus group data displayed a wide range of user issues associated with telehealthcare devices. There were 22 thematic categories to emerge from the data, however this paper focuses on the two themes associated with design characteristics and the lack of focus on end-user needs.

### Desirable device characteristics

The focus group participants and the expert interviewees addressed several factors relating to desirable device characteristics and these were viewed as essential to the uptake and ultimate usage of products.

#### Aesthetic appearance

For some, device aesthetics were clearly important particularly with wearable devices, “*just because you're older doesn’t mean you don’t want to look nice*” (Oxfordshire focus group participant). Some personal alarm users stated that they did not wear their devices because of the appearance of the device, but for others, this was not so important. One Bedfordshire focus group participant suspected that “*there’s a gender factor here*”, with the implication being that women may at times worry more about appearance than men. Nevertheless, the importance of device aesthetics was stated more strongly by the experts.

*“I think it's predominantly aesthetics, I mean most of the equipment, with the exception of a few, is turned out in a sort of clinical white box type structure” (Interviewee 015)*

Thus, some of the devices were seen as “*not particularly well designed, but I'm also talking about… the appeal, they're not very sexy*” (Interviewee 007). The experts linked aesthetic issues to stigmatisation and ultimate non-usage:

*…One of the issues is the ugliness of some telecare equipment. And this is a general issue for the market place responding to older people, as consumers to understand that they like anybody else would like to have something attractive in their home… What one tends to get is incredibly functional off-putting things, which have a big stigmatising label…*

*(Interviewee 006)*

For the experts the key was well-designed technologies, which people wish to use, and prevent stigmatisation. It was important for them not to make assumptions about end-user views on attractive devices, and what would suit an individual and their home environment, but to provide a range of suitable choices.

*…I think the technologies are still very clunky… There's nothing cool about them, and the choices. I've always taken the view that if your grandchild doesn’t want it then I don’t want it either… It all matters, and it's in their home. They don’t actually want the appearance of their home to change… And that means more choices…*

*(Interviewee 001)*

Another participant discussed the implications of poor aesthetics of pendant personal alarms, causing individuals to place the alarm under their clothes:

*Interviewee 010: Even on a very practical level… you may aspire to use it, but the fact they’ve buried it under six layers means they're not going to be able to use it if they're on the floor.*

*NC: Is that related to stigma, burying it under layers?*

*Interviewee 010: I think it is, yeah… There was an element of, especially when it came with all these other ugly bits of equipment that ruined the look of their house… I think that was a bit of an issue for people… who like to be quite well turned out and the idea of it looking a bit jarring with the way they usually like to dress and present themselves…*

The aesthetics of devices were viewed as increasingly important as the market “*is moving towards a more commercial retail model. And I think those issues will become more important as the shift continues… Again, with the same kind of thought really as how to make these products, not cool that's the wrong word, but appear normal [laughs], mainstream*” (Interviewee 017). Mainstreaming of technologies, like the activity monitors often referred to, through attractive aesthetics was viewed as essential.

#### Practicality and ease of use

All the study participants identified the need for good design of telehealthcare products. Good design elements were viewed as practicality, ease of use, efficiency, confidence inspiring and flexibility to adapt to changeable needs and an illness trajectory. The design of any device was required to inspire confidence in the following ways:

*…I think it's confidence that it will actually do what it says, what you want it to. I think it's confidence in understanding how it works, that you can use it. I think it's also confidence that you won't have someone come round and decry it…*

*(Interviewee 018)*

Other “good design” elements included the importance of interoperability, with supportive platforms to enable flows of data. Ease of use was particularly important for the focus group participants, “*I also think it's important that it's not technically too challenging for anybody who’s older*” (Bedfordshire focus group participant). Ease of use was also linked to intuitive technologies by the experts, which were perceived as well-designed products, which require minimal training to use:

*…Intuitive (devices) that's the most important thing… Design is actually really important. You know there were plenty of tablet computers before the iPad, but they never took off did they? Once they produced one that actually worked and was easy to work everyone had one. And I think that's true of these types of devices…*

*(Interviewee 021)*

The focus group participants contrasted ease of use as a concept to a general perception of device complexity. Many of the devices were seen as overly sophisticated:

*…What's beginning to disturb me a little bit is that as these devices you're putting round, are getting more and more complex, sophisticated, and multi-functional, there must be an equal multiple in the number of false alarms that you get. My worry is with this GPS and interaction, with all sorts of other functions as well, you might end up with relatives dashing up and down the M1 to see somebody who is perfectly all right, and in Tesco, but you think they’ve fallen downstairs. I'm just a bit worried if we’re not too careful… the scientists tend to get sort of carried away with their technology, and want to add more and more bells and whistles… That device (a mobile alarm, tracking device) worries me; I think you need at least a PhD in something or other before you master that one…*

*(Bedfordshire focus group participant)*

The experts often cited examples of poor design, particularly with the telecare devices, and there was some criticism levelled at the manufacturing companies in this regard as “*they sort of accept that it's being used and that's enough*” (Interviewee 004). For the focus group participants, the purpose of the respective devices available within the groups was not at all clear from their appearance, suggesting a lack of intuitive design.

The experts referred to examples of devices having to be have home adaptations “*bricolage*” to meet needs. This level of adaptation suggests that some devices are not meeting individual needs. One participant spoke of clients getting “*really creative with Sugru* 1 *and all sorts of things*” (Interviewee 018). For other devices that could not be successfully adapted/adjusted by the end-user the ultimate outcome was one of individuals asking for devices to be removed:

*…You know if it's not working right, for example, the bed sensors if they're not working right straight away the customer says* “take it out, I don’t want it, I don’t want it”*. We found a lot of the carers, family and relatives were saying* “it's okay we’ll adjust it, lets have a go”*. But no, the actual person using it didn’t want it at all…*

*(Interviewee 004)*

The importance of focusing on end-user needs and requirements was a clear recurrent theme within the data.

### The lack of focus on end-user needs

The expert participants in the study identified the essential requirement of focussing on end-user needs. This was often not evident in the examples shown. Manufacturers and suppliers were perceived at times as offering equipment that met with their own management needs, or preconceptions, rather than that of the end-user. Moreover it was argued that the specific needs of older people were not being successfully met. This was seen as being caused by viewing them as a discrete group, resistant to technology, rather than focusing on developing appealing and intuitive products that work for individuals irrespective of age.

*…We really do have to think in terms of a change in service paradigms to get away from the awful situation in which service providers are offering particular ranges of kit or equipment, and services that accord with their management needs, or accord with their particular preconceptions about how products should be designed and used to give them the data that they need, as opposed to actually thinking much more from the user or indeed the carer perspective, and thinking about what they need… One of the important things here is to escape from outside of the straightjacket of thinking that is around older people, and to think much, much more widely in terms of what you, I, or indeed an 18 year old might want to help them manage their diabetes, or their lifestyles, or their health…*

*(Interviewee 008)*

A County Council based participant went further by arguing that needs were not met as individuals are made to fit the equipment rather than the other way round:

*…We’re making the people fit the equipment that we can get, rather than having an issue for the service user and finding equipment to fit the user…*

*(Interviewee 020)*

Others echoed this in the sample. One said “*sometimes I think people have a great idea and then try and find a use for it, rather than understanding what is the problem*”. They have a “*clever idea and then try and fit people into it*” (Interviewee 018). This lack of devices to meet end-user needs and wants, was key in affecting uptake of devices.

*…Taking a kind of a mauve coloured, or whatever it is that the colour of these NHS plastic boxes and giving it to someone for their telehealth or their telecare solution is the least likely way of succeeding in deploying something. You know, at least if it looked like something that people had designed with the manufacturer, and felt right then it might have a better chance of being actually adopted, used, and left on display in someone’s front room…*

*(Interviewee 019)*

The interview participants identified that current products and their supply were not successfully meeting end-user needs. They identified that assumptions were made about end-user requirements and their usage of technology, particularly assumptions about the use of technology by older people. In the words of one:

*…The myth that we need to explode in this is citizens or patients resistance to technology… Most people are incredibly receptive and equally this nonsense that because you're 75 or 80 you can't use technology. There are some 75 year olds who cannot, and there are some 25 year olds who cannot, but it's not an age specific issue. It comes down to how your technology is designed, the look and feel of it, and more importantly the service model…*

*(Interviewee 025)*

Another participant spoke of these barriers as “*perceptions around people’s ability to use technology*” (Interviewee 014). This was associated with the need to provide “*confidence to patients that the use of these technologies doesn’t mean that it's taking away… contact when they need it, and care when they need it from their clinician, or carer, or whoever it might be*” (Inteviewee 014). Furthermore, where products do exist to meet needs experts emphasised “*the importance of getting it right first time in terms of taking time with the client, with the service user, and their family to ensure that they understand how to use the technology*” (Interviewee 006).

In relation to the identification of unmet user needs the experts called for greater user feedback. One participant highlighted the shortcomings of telecare base units and personal alarms to this end:

*…If you fall upstairs and your base unit is downstairs then that is just a complete and total waste of time. You know, people want to wear it (the personal alarm) in the garden… I think these things are incredibly challenging… But I think at least getting the user perception… having those discussions with people is a massively good start…*

*(Interviewee 013)*

An interpretation was made that the telecare side of the market was a statutory one. Therefore the manufacturers and suppliers had a “*pretty captive*” market and were already selling their devices in sufficient volumes, so user-involvement in design and development was not sought (Interviewee 022).

Despite the inherent challenges, the vast majority of the expert sample saw the need for end-user involvement in design and development of products as key. It was viewed as imperative i.e. something that should be more of a “standard principle” within device design and development. Although many recognised that this was the way things should be done it was noted: “*it's surprisingly often missed as a principle*” (Interviewee 006). Others spoke of it not being performed and one said “*you know these things are all being developed entirely in a vacuum, nobody ever asks anybody what they want*” (Interviewee 013).

User-centred design was viewed as an imperative underlying principle for design and development. Its success was seen as generating products “*people want rather than need*” (Interviewee 012). It was recognised as making commercial sense, but as alluded to above (Interviewee 013), it was viewed as something that could be difficult to achieve. This was because of the underlying difficulties in explaining product concepts without prototypes and the need for continual iteration with users:

*…I'm a strong supporter of involving users because at the end nobody can know better than them what they are ready to accept… More and more there is this kind of approach to… try to involve the user from the very beginning. Now you know that in reality that these kind of users you cannot just put them in front of a blank sheet and say “what would you like?” So fundamentally you have to develop prototypes, you need to have more caps in such a way that they can really express their opinion of something which is tangible that they can try and use… I think the development of products has to be an iterative process, which you try your first prototype, you get a feedback from the users, you improve it, and so on and so on…*

*(Interviewee 007)*

The need to specifically involve end-users, rather than professional users such as clinicians was also discussed. As one participant stated:

*…There has to be end-users involved because one of the problems there is, of course, is you can come up with an absolutely fantastic idea that the clinician really likes or loves to use in the clinic, but it's that interface… The interface that goes on with the end-user has to be correct…*

*(Interviewee 024)*

We also found considerable mistrust between various parties. This mistrust was not just directed at manufacturers and suppliers by user groups, county councils, research organisations and health focussed individuals but could be heard in all directions between the groups.

*…The main issue revolves around a very high degree of distrust between the various players. So player group one is the provider, which is generally NHS, though it should be local authority and is more and more becoming local authority. Group two are the clinicians. And group three are the actual patients. And of course all of those have to trust the supplier, but actually none of them do [laughs]… What we need is consensual collaborative solutions, not adversarial solutions…*

(Interviewee 023)

Careful consideration was also called for of who were the real end-users. There was discord over whether the users of a respective device were the formal or informal carers or professional users. A research based participant said: “*I mean I've heard manufacturers say this has tested really well with the users, and then when you drill down in to what they mean by users they mean carers. They don’t mean the end-user”* (Interviewee 010). Therefore, consideration of the respective users of a particular device and its systems required mapping so as to successfully involve all users:

*…I think an awful lot of technology that is out there at the moment has really not gone through proper user involvement in terms of design. And were it to, I think people would have to sit back and say* “well, who are all the users in this system?” *…Everybody that comes through my door I say the first question is, “who isn't this designed for?” And none of them can answer it…*

*(Interviewee 012)*

## Discussion

Digitisation of health systems should result in better health, better healthcare and lower cost [11] as exemplified by wearable technologies. In the view of the NHS England’s Medical Director this should enable better prediction, allowing earlier medical intervention and preventing unnecessary hospital admissions. But these systems and devices cannot be imposed on individuals. Usability requires that systems and device characteristics must include ease of use, intuitive design, and interoperability through to issues of aesthetics.

Problems with device usability were shown previously [12] in a study which tracked devices installed in the homes of individuals aged 60-98 over a 2 years period and found few met few participants’ needs. Some had been abandoned, a few deliberately disabled whilst others had been pragmatically customised by carers. Personal alarms are a classic example of poor usage and in our study, only one of the four personal alarm users wore her pendant all the time. This corresponds with a survey of personal alarm users in North East Scotland [13], where almost two-thirds of the respondents had never used it to summon help and most had never wanted to use it. Moreover, a significant minority (11%) had found themselves without their pendant when it was needed, this was because nearly one-third of the study cohort wore their alarm only some of the time, very occasionally or not all.

From a user perspective, our study revealed that the devices were not meeting the needs of end-users. Many devices were not aesthetically pleasing, non-intuitive and the majority of the focus group participants failed to recognise the purpose of the devices from their appearance alone. Of course, there are limitations to our study mainly associated with sample size. The small sample size for end-users (focus group participants) was an issue. However the focus groups themselves were relatively large to account for non-attendance in the older population, but only one participant did not attend on the day. Increasing the number within a focus group can improve the opportunities for obtaining a representative view to aid transferability of findings, but equally it may limit the active contribution of some participants. Whilst holding groups at different geographic locations assisted extrapolations from our findings, along with data from national experts, the small sample size of our study limits our ability to generalise. So too does the gender imbalance of one of the focus groups. Additionally, the fact that the focus groups participants were self-selecting may also limit the implications of our findings. Nevertheless our data demonstrates that without end-user involvement in device development there is likely to be poor uptake and usability.

These views must be addressed given the recognition placed by the literature and standard bodies that user involvement in the design and development process offers many benefits including safer and more usable devices [14]. More specifically to telecare alone, in a market analysis undertaken by Inventya [15] only 20% of the manufacturers engaged end-users themselves in the research and development phase of product development. Our experts highlighted the view that often people were made to adapt to the equipment rather than tailoring the system to their needs. Similarly a previous interview-based study [14] with 11 medical device manufacturers, revealed that the manufacturers were hesitant to involve users with the design and development process because of: perceived barriers to obtaining ethical approval; the speed at which such activity may be carried out; the belief that there is no need given the “all knowing” nature of senior health care staff and clinical champions; and a belief that effective results are achievable by consulting a minimal number of champions.

## Conclusions

Good design should intuitively inform users, and reflect what users need it to accomplish and in what context and setting devices are used [16]. Unfortunately, healthcare needs can be difficult to grasp and acknowledge both by the individuals themselves (as the focus group participants recognised) and by care professionals. Needs are highly individual and can change rapidly in the context of long-term conditions.

Our study outcome, showing a lack of user-centred design within telehealthcare product development is disappointing. If the recommendations about device systems being designed with the input of end-users [11] are to be achieved then the transfer of robust data between designers and older people is needed despite widely differing views about the understanding of appropriate products [17]. In addition end-users must be successfully identified rather than “proxy” users such as carers or professional users as the data clearly demonstrated that these other “proxy” users may have very different views, priorities and needs to the ultimate end-user. This is a finding that has been mirrored by other researchers in the area.

Moreover, if end-user needs are to be successfully met and user involvement addressed, the existing market focus needs to be challenged. Interviewees argued that the market was supplier rather than end-user driven, with manufacturers and suppliers dictating both the products and their supply. This has been found by other researchers who argue that there is a poor understanding of user needs with respect to telecare technologies, partly because the industry tends to be dominated by suppliers that are providing a technology-push rather than a demand-pull approach. In order to challenge this, the focus within the market must be reframed, so as to create a future marketplace that is driven by end-user needs.

## Abbreviations

Dhealth: Digital health
Ehealth: Electronic health
ICT: Information and Communication Technology
MHealth: Mobile health
NHS: National Health Service

## Footnotes

1 A mouldable glue that turns to rubber

## References

[1] He W, Goodkind DA, Kowal P, Reports IP (2016). An aging world:2015.

[2] Bureau USC: International Data Base world population by age and sex. https://www.census.gov/population/international/data/idb/worldpop.php. Accessed 29 June 2017.

[3] Blunt I, Bardsley BM, Dixon J. In: Trust TN, editor. http://www.nuffieldtrust.org.uk/publications Trends in emergency admissions in England 2004-2009; 2010. Accessed 29 June 2017.

[4] Collerton J, Davies K, Jagger C, Kingston A, Bond J, Eccles MP, Robinson LA, Martin-Ruiz C, von Zglinicki T, James OF (2009). Health and disease in 85 year olds: baseline findings from the Newcastle 85+ cohort study. BMJ.

[5] Ageing Britain: two fifths of NHS budget is spent on the over-65’s the Guardian https://www.theguardian.com/society/2016/feb/01/ageing-britain-two-fifths-nhs-budget-spent-over-65s. Accessed 29 June 2017.

[6] DALLAS Collaboration (2014). Self Care in the Digital age - new horizons for self care. A white paper.

[7] The NHS budget and how it has changed. The Kings Fund. https://www.kingsfund.org.uk/projects/nhs-in-a-nutshell/nhs-budget. Accessed 29 June 2017.

[8] Parliamentary Office of Science and Technology (2014). Telehealth and telecare.

[9] Braun V, Clarke V. Using thematic analysis in psychology. Qual Res Psychol. 2006;3(2):25.

[10] Glaser B. The constant comparative method of qualitative analysis 12(4): 436-445. Soc Probl. 1965;12(4):9.

[11] Wachter RM. Making IT work: harnessing the power of health information technology to improve care in England. https://www.gov.uk/government/publications/using-information-technology-to-improve-the-nhs/making-it-work-harnessing-the-power-of-health-information-technology-to-improve-care-in-england; 2016. Accessed 29 June 2017.

[12] Greenhalgh T, Wherton J, Sugarhood P, Hinder S, Proctor R, Stones R (2013). What matters to older people with assisted living needs? A phenomenological analysis of the use and non-use of telehealth and telecare. Soc Sci Med.

[13] Taylor A, Agamanolis S (2010). Service users’ views of a mainstream telecare product - the personal trigger. Proceedings of the 28th International Conference on human factors in computing systems, CHI April 10-15 2010; Atlanta, USA.

[14] Money AG, Barnett J, Kuljis J, Craven MP, Martin JL, Young T (2011). The role of the user within the medical device design and development process: medical device manufacturers’ perspectives. Med Inform Decis Mak.

[15] de Leonidas V, Bartosova R, Lewis E, AKTIVE consortium (2013). ALT market in the UK. AKTIVE Market Report: Initial Overview.

[16] Lehoux P, Saint-Arnaud J, Richard L (2004). The use of technology at home: what patient manuals say and sell vs. what patients face and fear. Sociol Health Illness.

[17] Buckle P, AKTIVE Consortium (2014). Human factors that influence the performance of the Telecare systems. AKTIVE working paper 7.

